# Case report: A STAT1 gain-of-function mutation causes a syndrome of combined immunodeficiency, autoimmunity and pure red cell aplasia

**DOI:** 10.3389/fimmu.2022.928213

**Published:** 2022-08-29

**Authors:** Yifan Xie, Fenli Shao, Juan Lei, Na Huang, Zhidan Fan, Haiguo Yu

**Affiliations:** ^1^ Department of Rheumatology and Immunology, Children’s Hospital of Nanjing Medical University, Nanjing, China; ^2^ State Key Laboratory of Pharmaceutical Biotechnology, Department of Biotechnology and Pharmaceutical Sciences, School of Life Sciences, Nanjing University, Nanjing, China; ^3^ Department of Pediatric Nephrology, The Second Affiliated Hospital of Nanjing Medical University, Nanjing, China

**Keywords:** STAT1 gain-of-function mutation, immunodeficiency, autoimmunity, pure red cell aplasia, STAT1 phosphorylation

## Abstract

Inherited autosomal dominant gain-of-function (GOF) mutations of signal transducer and activator of transcription 1 (STAT1) cause a wide range of symptoms affecting multiple systems, including chronic mucocutaneous candidiasis (CMC), infections, and autoimmune disorders. We describe a rare case of STAT1 mutation with recurrent CMC, lung infections, and anemia. According to the whole-exome sequencing (WES), the patient was genetically mutated in STAT1 GOF (c.854A>G, p.Q285R), and bone marrow biopsy suggested pure red cell aplasia (PRCA). As a functional verification, STAT1 levels and phosphorylation (p-STAT1) of peripheral blood mononuclear cells (PBMCs) following IFN-γ stimulation in STAT1 GOF patient was higher than in the healthy control. Combination therapy of blood transfusion, antimicrobials, intravenous immunoglobulin, methylprednisolone, and the Janus Kinase (JAK) specific inhibitor ruxolitinib was used during treatment of patients. The patient also received a hematopoietic stem cell transplant (HSCT) to help with infections and anemia. This is the first reported case of STAT1 GOF disease complicated with PRCA. This complication might be attributed to immune disorders caused by STAT1 GOF. Furthermore, ruxolitinib may be a viable therapeutic option before HSCT to improve disease management.

## Introduction

Signal transducer and activator of transcription 1 (STAT1) regulate cell proliferation, growth, metabolism, differentiation, and apoptosis as a central transcription factor in Janus Kinase-Signal transducer and activator of transcription (JAK-STAT) signaling. Besides, STAT1 mutations probably have a marked impact on the host defense against infections through disturbed T helper cell responses (1). In response to stimulation of extracellular receptors, activated JAK gives rise to STAT1 phosphorylation. STAT1 mutations have been increasingly found in patients since heterozygous pathogenic variants in STAT1 were first described as a genetic etiology of chronic mucocutaneous candidiasis (CMC) in 2011 (2). To date, as many as 105 STAT1 gain-of-function (GOF) mutations have been identified in above 400 patients worldwide, including 65 recurrent mutations (3).

STAT1 GOF mutations often underlie an autosomal dominant trait. The majority of STAT1 GOF mutations are found in the coiled-coil domain (CCD) and DNA-binding domain (DBD), with the CCD alone accounting for half of the cases (4). The pathogenesis of STAT1 GOF is associated with increased p-STAT1 levels and STAT1-dependent cellular responses. Different STAT1 GOF mutations may have different mechanisms of the elevated p-STAT1 levels, such as increased transcription, impaired dephosphorylation, or some other regulatory pathways. Therefore, increased protein expression of resting and stimulated STAT1 might serve as a direct screening method for STAT1 GOF mutations (5).

CMC is a primary immunodeficiency disease characterized by recurrent and persistent mucocutaneous lesions caused by *Candida albicans*, affecting the oral or genital mucosae, nails, and skin (6). Accumulated data indicates that the IL-17 cytokine is essential for the host’s defense against mucosal infection (7). Th17 cell deficiency triggered by STAT1 GOF mutations results in the emergence of CMC (8). Heterozygous STAT1 GOF mutations can account for at least 50% of genetic CMC cases (9). Besides CMC, STAT1 GOF mutations showed a variety of infectious phenotypes and can cause host susceptibility to viruses and extracellular or intracellular bacteria in addition to *Candida albicans (* 10). Moreover, these patients are more likely to develop various types of autoimmune diseases including hypothyroidism autoimmune cytopenia, vitiligo/psoriasis/alopecia, diabetes mellitus, and systemic lupus erythematosus (SLE), compared to the general population. Some may also suffer aneurysms and an increased risk of carcinomas (6, 10). The presence of these latter complications leads to a poor outcome. However, the broad clinical characteristics and outcomes of patients with STAT1 GOF remain unknown.

We herein report a child diagnosed with STAT1 GOF mutation accompanied by life-threatening symptoms of infection and anemia. As far as we know, this case represents the first report of pure red aplastic anemia reported in association with such a genetic defect.

## Case presentation

In 2020, an 8-year-old girl was admitted to our hospital with a cough with sputum, fatigue, and anemia. She was diagnosed with suspected SLE at the age of 1 year old but received no standard treatment. She received intravenous immunoglobulin (IVIG) treatment several times in the past. The patient suffered recurrent lung infection and oral *Candida* infection.

Physical examination on admission revealed edema of the face; pallor; hemorrhagic rash on the face and legs; thrush; superficial lymphadenopathy in the neck and axilla; a few moist rales in both of her lower lungs; hepatosplenomegaly; and no bilateral lower limb edema. The child had high C-reactive protein (CRP), rapid erythrocyte sedimentation rate (ESR), low complement [C3 0.619g/L (reference range 0.88~2.01g/L), C4<0.0673g/L (reference range 0.16~0.47g/L)] and elevated IgG 16.8g/L (reference range 7.91~13.07g/L). Autoantibody spectrum revealed that anti-nuclear antibody (ANA) was positive, while anti-SS-A, anti-SS-B, anti-dsDNA antibody, and anti-SM antibody were negative. Blood routine examination revealed a low red blood cell count of 1.84×10^12/L (reference range 3.5~5.5×10^12/L) and hemoglobin level of 56g/L (reference range 110~160g/L). High serum total bilirubin composed mainly of indirect bilirubin was detected, accompanied by a positive Coombs test. In both lungs, a chest CT revealed patchy and striped shadows. Multiple enlarged lymph nodes in the mediastinum and axilla; enlargement of liver and spleen; a small amount of effusion in the pelvis. Color Doppler echocardiography indicated pericardial effusion. Ultrasound showed multiple cervical lymph node enlargements on both sides and no obvious occupying lesions in the thyroid.

To treat anemia, the patient received a red blood cell transfusion. She was treated with standard antibiotics combined with methylprednisolone (1mg/kg iv q12h) and the antifungal fluconazole due to her obvious hemolysis and multiple infections. At the same time, the girl received IVIG 1mg/kg/day for 2 days to control hemolysis and infection. She was also given an immunosuppressant mycophenolate mofetil (MMF), but her hemoglobin concentration continued to drop. Meanwhile, she had hypothyroidism and was taking levothyroxine sodium tablets to treat it. Nevertheless, the child was re-admitted to our department several times due to repeated lung infections and anemia.

**Figure 1:**
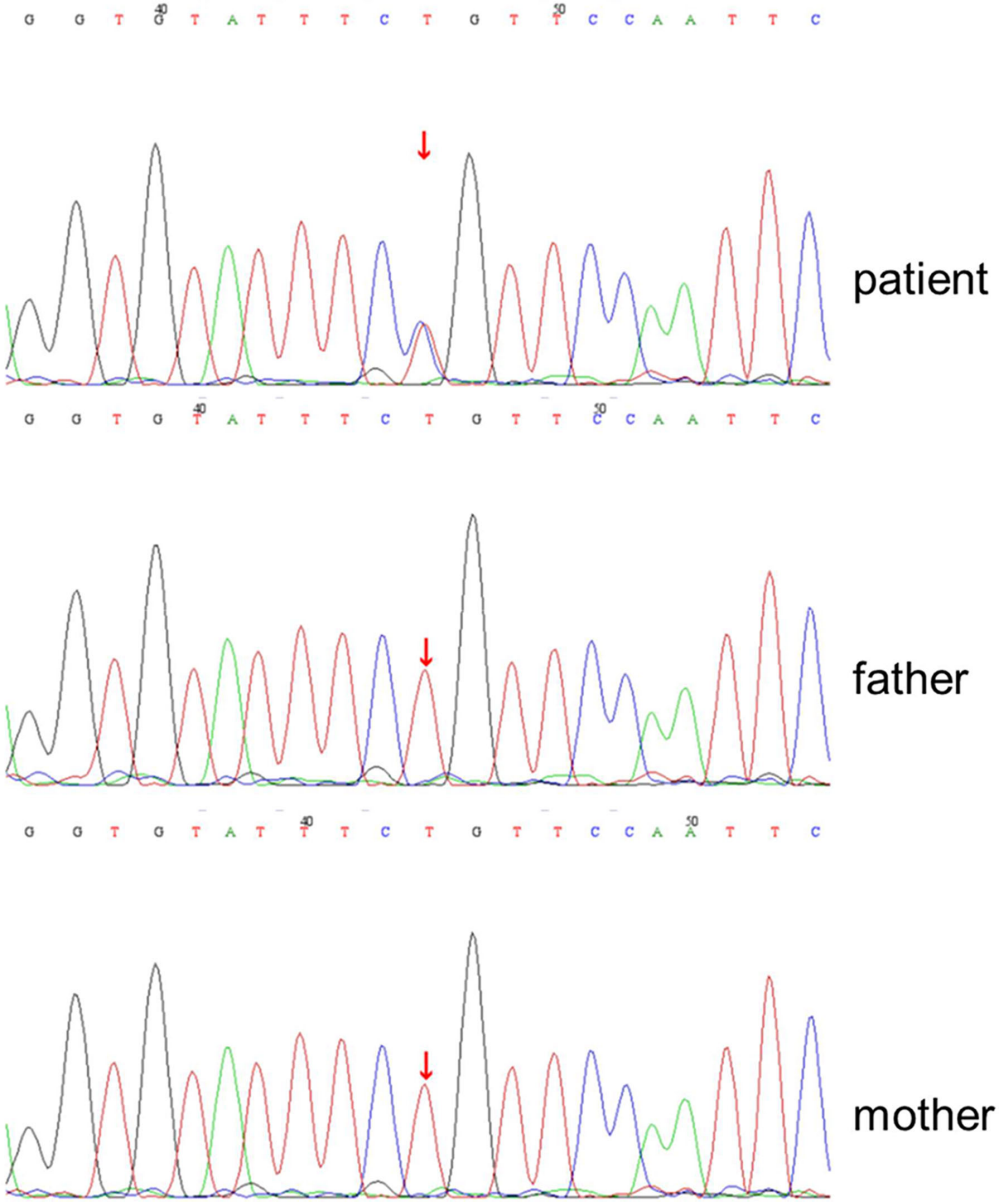
*De novo* heterozygous STAT1(c.854A>G) leading to p.Q285R amino acid change in STAT1 gene of the patient. DNA chromatogram of the patient and his parents (wild-type) was detected by whole-exome sequencing.

This girl was suspected of having primary immunodeficiency based on her clinical course and prior medical history. Whole-exome sequencing (WES) of this patient revealed that the STAT1 gene changed from adenine to guanine at no.854 nucleotide (c.854A>G), resulting in the change of amino acid 285 from glutamine to arginine (p.Q285R). The mutation was the first to occur in our patient, and neither of her parents was influenced (**Figure 1**).

**Figure 2:**
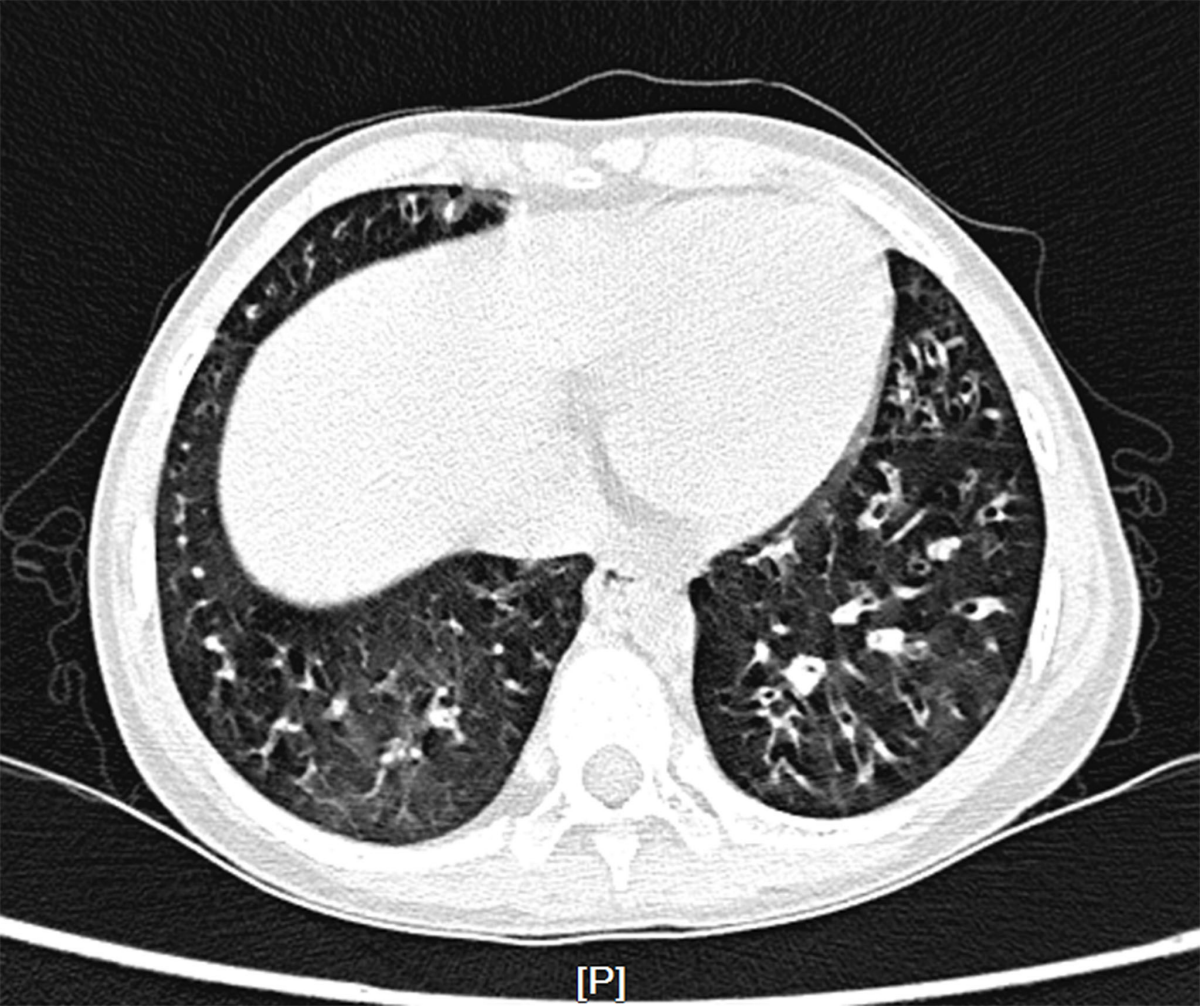
Chest computed tomography scan. Multiple bronchiectasis were randomly distributed in the bilateral lobe.

The patient was started on therapy with oral JAK inhibitor ruxolitinib at a dose of 5mg twice daily following confirmation of diagnosis for 4 months, then the dosage was reduced to 5mg once daily as maintenance therapy, and cotrimoxazole was also added to prevent fungal infection. After 6 months of treatment, the Coombs test became negative and hemolysis was under control, but anemia still occurred repeatedly. Reticulocyte count was gradually reduced to 1.1×10^9/L (reference range 24~84×10^9/L), indicating the child’s poor hematopoietic function. Further bone marrow morphology test and biopsy indicated a decline in bone marrow erythrocytes [erythrocytes 0.4% (reference range 15~25%), granulocytes/erythrocytes=158:1(reference range 2~4:1)], leading to the diagnosis of pure red cell aplasia (PRCA) (**Supplementary Figure S1**). In addition, due to repeated lung infections, the patient developed bronchiectasis (**Figure 2**).

**Figure 3:**
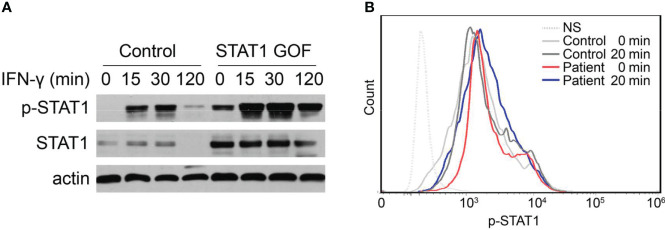
Enhanced STAT1 and p-STAT1 expression in the GOF patient. **(A)** Evaluation of STAT1 and p-STAT1 protein level in the GOF patient and healthy control by immunoblotting, at rest, 15′, 30′, 120′ after IFN-γ stimulation. **(B)** Patient and healthy control p-STAT1 level 20min after IFN-γ stimulation, as measured by flow cytometry.

To assess the impact of the mutation on STAT1 function, we detected STAT1 expression and phosphorylation response of the patient’s peripheral blood mononuclear cells (PBMCs) to IFN-γ by flow cytometry and immunoblot. When compared to additional healthy controls, Western blot analysis confirmed that IFN-γ could induce abnormally high STAT1 expression and phosphorylation in the patient’s PBMCs (**Figure 3A**). In addition, flow cytometry was used to assess the response of p-STAT1 to IFN-γ stimulation in whole blood, and p-STAT1 levels were higher in STAT1 GOF patients (**Figure 3B**).

Due to her rapid deterioration, the patient received a hematopoietic stem cell transplant (HSCT), but the therapeutic effect was not satisfactory. The patient developed gastrointestinal bleeding and severe pulmonary infection with consolidation.

## Timeline

**Figure 4:**
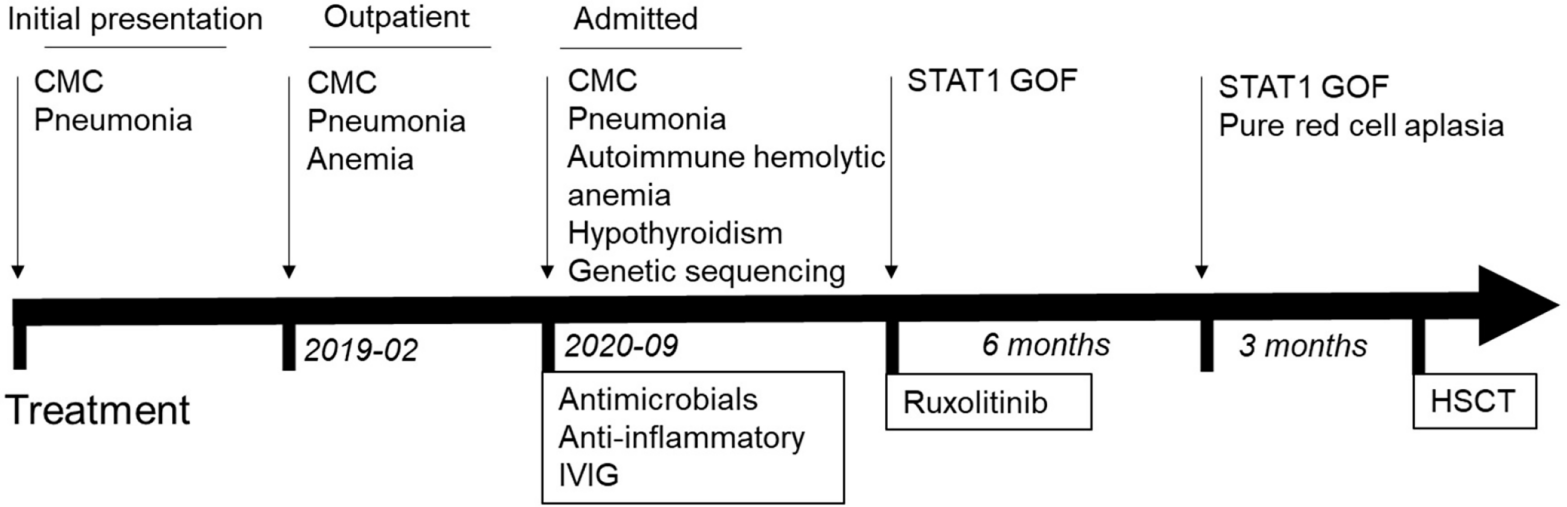
Timeline of clinical events and treatment strategies. HSCT, hematopoietic stem cell Transplant.

**Figure 4** depicts the timetable.

## Discussion

We describe a pediatric patient with a pathogenic STAT1 GOF variant presenting as recurrent anemia and infection. The initial presentation of this case was recurrent CMC and pulmonary infection, accompanied by severe anemia. Several of the patient’s features were suggestive of SLE, including effusion in the pelvis, hemolytic anemia, low complement, and ANA (+). In our opinion, this could also be an autoimmune SLE-like presentation of STAT1 GOF. The symptoms of infection and hemolysis were controlled by comprehensive treatments of antimicrobials, anti-inflammatories, immunosuppression, IVIG, and ruxolitinib.

Patients with heterozygous STAT1 GOF mutations frequently exhibit CMC, immunodeficiency, and autoimmune manifestations. In pediatric patients with STAT1 GOF, the most common symptom is a fungus infection. A large percentage of patients initially presented with CMC or sinopulmonary infections. CMC is among the wide spectrum of infections caused by severe T-cell deficiencies (3). As the disease progresses, patients develop many complications, including bacterial infection, lower respiratory tract infection, autoimmune thyroid disease, and others (10). It is difficult to infer this congenital disease from routine or immunological examination results. Accordingly, STAT1 GOF mutations can only be confirmed by gene sequencing.

The hyperactivity of STAT signaling leads to enhanced STAT1 function, which inversely impairs the production of STAT3-dependent cytokines (11). The inhibition of Th17 differentiation was caused by a loss of functional balance of STAT1/STAT3 signaling. The patient has higher levels of total STAT1 and p-STAT1 protein after stimulation. Indeed, a recent study suggests that STAT1 hyperphosphorylation in patients results not only from impaired dephosphorylation but also from an increase in total STAT1 (5).

Oral therapy with ruxolitinib inhibits JAK activation and represses STAT1 hyperresponsiveness in patients (12). Immunosuppression with the inhibitor normalizes autoimmunity while also restoring host defense (13, 14). It was clinically reflected in this patient by a significant improvement in CMC and autoimmune hemolytic anemia. However, early studies show that CMC is prone to relapse after treatment is discontinued and long-term (>12 months) usage of ruxolitinib is required to sustain the effect (15). To avoid potential side effects such as serious infections and cancer, the dose must be carefully titrated, as with other immunomodulatory and immunosuppressive medications. Another study reports that ruxolitinib therapy can better manage disease before HSCT, and possibly improve its prognosis (16, 17). Although it remains uncertain about the safety and efficacy of prolonged treatment with ruxolitinib, immunoglobulin replacement therapy, and JAK inhibitors are attractive alternatives in STAT1 GOF patients.

In addition, HSCT has been used to treat STAT1 GOF patients with recalcitrant infections or severe autoimmune disorders. However, overall survival after transplantation was reported to be 40%. The immune phenotype of STAT1 GOF mutations increases the transplant-related risk for severe infections, graft versus host disease, and gastrointestinal and pulmonary bleeding (18). Considering its high rate of fatal complications and unfavorable outcomes, HSCT is currently not the first choice for such a disease.

Several patients with STAT1 GOF mutations had both CMC and severe autoimmune diseases. STAT1 GOF mutations facilitate STAT1 hyper-phosphorylation and amplify transcription of IFN-stimulated genes, which result in autoimmunity. Surprisingly, the child’s anemia may be attributed to two main etiological factors: bone marrow hematopoietic dysfunction caused by PRCA and autoimmune hemolysis. The autoimmune disorder was critical to the pathogenesis of autoimmune hemolysis. Nevertheless, there is a crucial question. Was STAT1 GOF mutation of the patient related to her PRCA? Previous reports have suggested that aplastic anemia might be associated with STAT1 GOF variants (12). But the cause of PRCA in STAT1 GOF is not yet clarified.

It is widely agreed that immune-mediated erythropoietic exhaustion is critical for the pathogenesis of PRCA in autoimmune diseases. Autoimmune diseases are distinguished by abnormal CD4+ and CD8+ T cell activation. Recent studies have identified that STAT1 GOF mutation triggers a positive feedback loop between CD8+ T cells and IFN-responsive monocyte to increase the risk of aplastic anemia. Further, the CD8+ T cell activated by cytokines targets and destroys hematopoietic stem cells (19, 20). We reasoned that this patient’s PRCA might be caused by immune attacks on hematopoietic cells, which disrupted erythroid differentiation (21). T-cell-mediated or autoantibody-dependent immunologic processes may be responsible for this (22). Another possibility is that IFN-γ reduces both the life span and formation of erythrocytes in patients with diseases involving chronic immune activation (23, 24). Despite this, causes of medications or infections cannot be completely ruled out. Additionally, it’s still possible that PRCA was idiopathic in this case.

The novelty of this case report lies in describing the possible connection between PRCA and the STAT1 GOF mutation. However, the potential pathogenic mechanisms for PRCA driven by STAT1 GOF mutation requires further investigations.

## Materials and methods

### PBMCs isolation

Ficoll centrifugation was used to isolate PBMCs from the patients and healthy donor blood. Resuspended PBMCs were cultured in an RPMI medium containing 10% fetal bovine serum (Gibco).

### Immunoblotting assays

PBMCs were incubated with or without IFN-γ (100ng/ml, Peprotech, 300-02) for 15, 30, or 120min. Cell proteins were extracted by lysis buffer containing protease and phosphatase inhibitors (Sigma Aldrich). The lysates were incubated and centrifuged at 4°C. A Micro BCA protein assay kit (Pierce, Thermo) was used to determine protein concentration. Proteins were separated on SDS-PAGE gels and transferred to PVDF membranes. The membranes were blocked in TBS-T (0.1% Tween 20 in TBS) containing 5% defatted milk for 1h at room temperature and probed with primary antibodies against phospho-STAT1 (Tyr701) and STAT1 (Cell Signaling Technologies). The membranes were washed and incubated with HRP-tagged secondary antibodies. As an internal standard control, β-actin (Abmart, M20010) was used. Quantification of intensity was analyzed by Image J software.

### Phospho-flow

PBMCs were stimulated w/wo IFN-γ (100ng/ml) for 20min. For intracellular staining, the harvested cells were fixed and permeabilized with intracellular staining Buffer Set according to the manufacturer’s instructions (eBioscience). Cells were washed and incubated with anti-phospho-STAT1 (Cell Signaling Technologies, #9167) and anti-Rabbit-Alexa Fluor 488 (abcam, ab150077), respectively. The data were collected with FACSaria II (BD Biosciences) and analyzed by FlowJo.

## Data availability statement

The original contributions presented in the study are included in the article/**Supplementary Material**. Further inquiries can be directed to the corresponding authors.

## Ethics statement

The studies involving human participants were reviewed and approved by Children’s Hospital of Nanjing medical University. Written informed consent to participate in this study was provided by the participants’ legal guardian/next of kin.

## Author contributions

YX collected and interpreted the case report data and wrote the original draft. FS has performed the experiments. JL and NH participated in data collection and analysis. HY and ZF lead the multidisciplinary care of the patients and reviewed the manuscript. All authors contributed to the article and approved the submitted version.

## Funding

Jiangsu Province’s Natural Science Foundation provided funding for this research (grant No. M2021080).

## Conflict of interest

The authors declare that the research was conducted in the absence of any commercial or financial relationships that could be construed as a potential conflict of interest.

## Publisher’s note

All claims expressed in this article are solely those of the authors and do not necessarily represent those of their affiliated organizations, or those of the publisher, the editors and the reviewers. Any product that may be evaluated in this article, or claim that may be made by its manufacturer, is not guaranteed or endorsed by the publisher.

## Supplementary material

Supplementary Figure 1 Slide of the bone marrow biopsy. Decline in bone marrow erythrocytes.
