# Supplementary figures and images for: Case report: A STAT1 gain-of-function mutation causes a syndrome of combined immunodeficiency, autoimmunity and pure red cell aplasia
Source: Front Immunol. 2022 Aug 29;13:928213. doi: 10.3389/fimmu.2022.928213 (PMC9464931; doi:10.3389/fimmu.2022.928213)

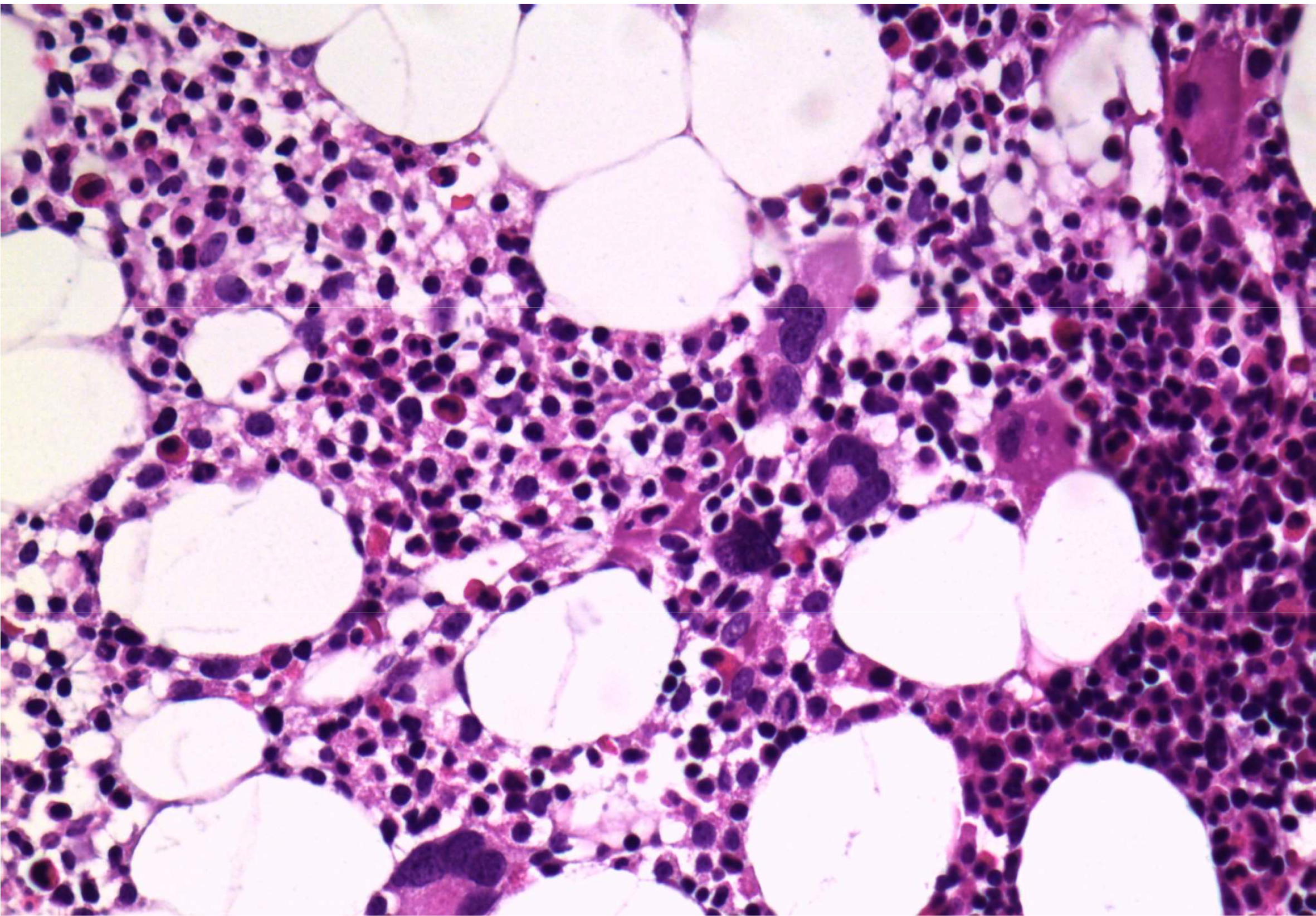

Supplement: Supplementary Figure 1 — Slide of the bone marrow biopsy. Decline in bone marrow erythrocytes. [file Image_1.tif]
